# The Pattern and Influencing Factors of Opioid-Prescribing Behavior Among Emergency Physicians: A Cross-Sectional Study in the Western Region of Saudi Arabia

**DOI:** 10.7759/cureus.48502

**Published:** 2023-11-08

**Authors:** Hamdan Alrajhi, Sawsan Hanafi, Malak BinShihon, Mahmoud Halawani

**Affiliations:** 1 Emergency Medicine, King Fahad Armed Forces Hospital, Jeddah, SAU; 2 Emergency Medicine, King Abdulaziz Medical City, Jeddah, SAU; 3 Emergency Medicine, King Salman Medical City, Madinah, SAU

**Keywords:** pain management, addiction, emergency medicine, prescribing behavior, opioids

## Abstract

Background

In the last two decades, drug overdose has globally become a major player in patients’ morbidity and mortality events. Opioids, in particular, have been always the main part of this equation in different communities as they correspond, for instance, to one-third of poisoning deaths in The United States of America (USA).

Aim

This study aimed to measure the variation in opioid-analgesia (OA) prescription behavior among emergency medicine (EM) physicians working in different hospitals in the Western Region of Saudi Arabia.

Subjects and methods

This is a cross-sectional study conducted among EM physicians in the Western Region of Saudi Arabia. A self-administered questionnaire was distributed among EM physicians using an electronic online survey. The questionnaire includes basic demographic characteristics and a 22-item questionnaire to assess opioid-prescribing behavior.

Results

A hundred and fifty-nine physicians took part in the study (male 61.6% vs female 38.4%). Of them, 59.7% were aged 23-30 years old, and junior residents constituted 35.1%. The factors that were associated with the most variable behavior were being aged 36-40 years old (p<0.001) and having more than 10 years in practice (p=0.007). The highest self-rated determinant factors were the apparent level of patients’ distress, types of medications that were given, physicians’ concerns about side effect profiles, patients’ diagnoses, and pain scores.

Conclusion

EM physicians demonstrated an overall comparable prescribing behavior. Progression of physicians’ age and years of practice both significantly affected our participant behavior. The highest self-rated prescribing factors were patients’ distress level and the previously given medications. Further research is needed in order to implement better practical guidelines.

## Introduction

In the last two decades, drug overdose has globally become a major player in patients’ morbidity and mortality events. Opioids, in particular, have been always the main part of this equation. Different communities experience high rates of abusing these drugs. For instance, opoid medications corresponded, in 2013, to one-third of poisoning deaths in The United States of America (USA), with a 4-fold increase from 1999 to 2013 [1]. A considerable number of emergency department (ED) visits is related to opioid use, with 420,040 cases of opioid analgesia (OA) overdose estimated in the year 2011 [2]. As per Cantrill et al., with different levels of cases’ complexities, most opioid prescriptions have been based on clinical experience rather than evidence-based guidelines [3]. Moreover, focused education about opioid administration is deficient in most of the four-year emergency medicine residency training programs (EMRTP) in Saudi Arabia (SA) in addition to the programs of the US [4, 5]. This, in turn, has motivated the trainees to adopt their own instructors’ prescribing behaviors [6]. As a result, there has been a high variability of opioid-prescribing patterns among EM residents based on their level of training and, surprisingly, in between the residents who are at the same level of training [7, 8]. To our knowledge, this area of research has not been previously studied among EM physicians in Saudi Arabia. Through this cross-sectional study, we aim to reach a general overview of the different patterns and influencing factors of opioid prescribing among EM physicians working at the hospitals of the Western Region (WR) in SA.

## Materials and methods

The opioid-prescribing behavior has been assessed by using a 22-item questionnaire with five Likert scale categories. The answer options range from “strongly disagree” which is coded as one to “strongly agree” which is coded as five.

Descriptive statistics were summarized as numbers, percentages, mean, and standard deviation. The differences in the score of behavior according to the socio-demographic characteristics of physicians were calculated using the one-way analysis of variance (ANOVA) test and independent-sample t-test. Post hoc analysis was subsequently performed to determine the multiple differences of variables by using the Tukey honestly significant difference (HSD) test. The normality test was performed using the Shapiro-Wilk test. A p-value of 0.05 was considered statistically significant. The data were analyzed using Statistical Packages for Social Sciences (SPSS) version 26 (Armonk, NY: IBM Corp, USA).

## Results

A hundred and fifty-nine physicians responded to our survey. One variable parameter was “practice,” by which we mean the total years of clinical work in the field of EM whether as a general practitioner, resident, or consultant. Junior residents are those who are at the first and second levels (years) of training whereas senior residents are those at the third and fourth levels. As described in (Table 1), most of the study population falls within the age group of 23-30 years old (59.7%), with males being dominant (61.6%). The majority had 13-16 shifts per month. One-third of the physicians (33.3%) had one to two years in practice and 35.1% were junior residents.

**Table 1:** Demographic and professional characteristics of the physicians (n=171)

Study variables	Sub-Groups	N (%)
Age Group	23 – 30 years	95 (59.7%)
	31 – 35 years	27 (17.0%)
	36 – 40 years	19 (11.9%)
	41 – 50 years	15 (09.4%)
	51 – 60 years	03 (01.9%)
Gender	Male	98 (61.6%)
	Female	61 (38.4%)
Number of shifts per month	≤12 shifts	12 (07.5%)
	13 – 16 shifts	119 (74.8%)
	>16 shifts	28 (17.6%)
Years of Practice	1 – 2 years	53 (33.3%)
	3 – 4 years	38 (23.9%)
	5 – 10 years	34 (21.4%)
	>10 years	34 (21.4%)
Level of Practice	Junior residents	60 (35.1%)
	Senior residents	43 (25.1%)
	Staff physicians	12 (07.1%)
	Consultants	56 (32.7%)

On the pre-mentioned scale of one to five, providers showed some variability in the self-judged importance of different factors affecting their decision to prescribe opioids (Table 2). The top five highest-rated elements were detected as follows (mean ± SD): the patient’s apparent level of distress (4.36 ± 0.79), type and amount of medications that were already given to control the pain (4.29 ± 0.84), physician’s concern about the possible side effects (4.08 ± 0.97), diagnosis thought to be the cause of pain (4.03 ± 0.94), and the patient’s reported pain score (3.99 ± 0.89). On the other hand, the lowest-rated factor was “my prescribing behavior of opioids is affected by my friends and family's experience with these medications” (1.88 ± 1.05). Based on the different levels of practice, attending physicians were more confident in identifying the OA-addict patients (p=0.099) whereas resident physicians are the least capable of judging the addiction signs. On another parameter, residents’ decisions were the most affected by patients’ specific requests for opioids (p=0.056).

**Table 2:** Assessment of opioid-prescribing behavior among emergency medicine (EM) physicians (n=171)

Behavior statement	Overall Mean ± SD	Residents Mean ± SD	Practitioners Mean ± SD	Consultants Mean ± SD	p-value ^§^
Diagnosis thought to be the cause of pain	4.03 ± 0.94	4.04 ± 0.95	3.82 ± 1.17	4.06 ± 0.86	0.732
The patient’s reported pain score	3.99 ± 0.89	4.06 ± 0.89	4.00 ± 1.00	3.83 ± 0.88	0.354
Vital signs and physical exam findings	3.97 ± 1.06	4.02 ± 1.01	4.27 ± 1.01	3.81 ± 1.16	0.340
The patient’s apparent level of distress	4.36 ± 0.79	4.43 ± 0.76	4.27 ± 0.79	4.23 ± 0.86	0.329
The patient’s age, gender, or nationality	2.50 ± 1.20	2.42 ± 1.22	2.82 ± 1.54	2.58 ± 1.09	0.488
Laboratory or imaging results	2.64 ± 1.18	2.49 ± 1.09	3.09 ± 1.22	2.83 ± 1.29	0.103
Patient’s opioid prescription history and reputation of likely abuse/addiction	3.28 ± 1.15	3.26 ± 1.19	2.64 ± 1.12	3.46 ± 1.05	0.099
Documented history of proven substance abuse/dependence	3.64 ± 1.11	3.62 ± 1.14	3.09 ± 1.22	3.81 ± 1.00	0.146
Patient’s other current medications	3.55 ± 1.00	3.59 ± 0.97	3.82 ± 0.98	3.42 ± 1.07	0.412
Patient’s specific request for opioids	2.96 ± 1.31	3.13 ± 1.28	2.27 ± 1.01	2.77 ± 1.37	0.056
Patient’s overall satisfaction	3.33 ± 1.11	3.41 ± 1.14	3.27 ± 1.10	3.17 ± 1.04	0.452
Type and amount of medications that were already given to control the pain	4.29 ± 0.84	4.28 ± 0.89	4.18 ± 0.98	4.33 ± 0.69	0.851
Your concern about the possible side effects	4.08 ± 0.97	4.08 ± 1.01	4.09 ± 0.94	4.08 ± 0.92	0.999
Your concern about promoting addiction	3.17 ± 1.30	3.20 ± 1.38	3.27 ± 1.10	3.08 ± 1.18	0.848
Your concern about if the patient is “doctor shopping	3.11 ± 1.26	3.07 ± 1.32	2.82 ± 1.33	3.25 ± 1.10	0.527
Your concern about the non-medical use of the medications	3.06 ± 1.29	3.02 ± 1.32	2.91 ± 1.30	3.19 ± 1.27	0.704
ED providers are possibly a significant source of opioid medications that are used illegally	2.53 ± 1.24	2.58 ± 1.27	2.27 ± 1.10	2.48 ± 1.20	0.700
I can accurately identify patients who are doctor shopping	2.99 ± 1.00	2.89 ± 1.00	3.09 ± 0.94	3.19 ± 1.00	0.228
I can identify patients who are addicted to opioids	3.26 ± 1.01	3.14 ± 1.02	3.18 ± 1.08	3.52 ± 0.92	0.095
I would rather over-prescribe opioids and risk illegal use, than under-treat patients’ pain	3.35 ± 1.21	3.40 ± 1.24	3.36 ± 1.12	3.25 ± 1.18	0.780
My prescribing behavior of opioids is affected by my friends and family's experience with these medications	1.88 ± 1.05	1.86 ± 1.08	1.55 ± 0.82	2.00 ± 1.03	0.414
My prescribing behavior of opioids is affected by the prescribing culture of my emergency department and colleagues	2.61 ± 1.23	2.72 ± 1.23	2.00 ± 1.34	2.52 ± 1.17	0.152

The socio-demographic characteristics of the physicians played a clear role in the variability of responses and agreement, or disagreement, with the survey statements. We found that a higher general agreement was more associated with the age group (F=6.762; p<0.001) and those who had more than 10 years in practice (F=4.235; p=0.007). However, the gender (p=0.185), number of shifts per month (p=0.282), and level of practice (p=0.807) did not constitute a statistically significant variation in responses (Table 3).

**Table 3:** Variability of responses and statement agreement according to the physicians’ demographic and professional characteristics (n=171)

Factor	Sub-groups	Agreement Score (110) Mean ± SD	F/T-test	p-value
Age group ^a^	23 – 30 years	71.1 ± 7.99	F=6.762	<0.001
	31 – 35 years	70.5 ± 9.56		
	36 – 40 years	80.3 ± 11.6		
	>40 years	75.5 ± 9.30		
Gender ^b^	Male	73.4 ± 9.64	T=1.331	0.185
	Female	71.3 ± 8.86		
Number of shifts per month ^a^	≤12 shifts	76.3 ± 11.4	F=1.278	0.282
	13 – 16 shifts	72.0 ± 8.70		
	>16 shifts	73.5 ± 11.0		
Years of practice ^a^	1 – 2 years	71.0 ± 7.71	F=4.235	0.007
	3 – 4 years	70.2 ± 8.48		
	5 – 10 years	73.1 ± 10.2		
	>10 years	77.1 ± 10.5		
Level of practice ^a^	Junior residents	72.4 ± 8.49	F=0.326	0.807
	Senior residents	73.1 ± 10.7		
	Staff physicians	70.1 ± 7.99		
	Consultants	72.9 ± 9.72		

Further analysis of the age and years of practice was done in post hoc analysis (Table 4). The mean difference of the behavior score between 23-30 years old versus 36-40 years old was statistically significant (p<0.001). Also, there was a significant difference in the mean behavior score between 31-35 years old and 36-40 years old (p=0.002). Regarding years of practice, a significant mean difference was observed between physicians who had one to two years in practice versus physicians who had more than 10 years in practice (p=0.014). In addition, there was a significant mean difference in behavior scores between physicians who had three to four years in practice versus physicians who had more than 10 years in practice (p=0.009).

**Table 4:** Post-hoc analysis to determine the multiple differences in behavior score according to the age group and years of practice (n=159) *the mean difference is significant at the 0.05 level.

By Age		Mean Difference (I-J)	Std. Error	Sig.	95% Confidence Interval		
(I) Age group	(J) Age group				Lower Bound		Upper Bound
23-30 years old	31-35 years old	.55517	1.94004	.992	-4.4834		5.5937
	36-40 years old	-9.18947^*^	2.23557	.000	-14.9955		-3.3834
	41-50 years old	-4.42632	2.28674	.218	-10.3652		1.5126
31-35 years old	23-30 years old	-.55517	1.94004	.992	-5.5937		4.4834
	36-40 years old	-9.74464^*^	2.66376	.002	-16.6627		-2.8265
	41-50 years old	-4.98148	2.70684	.259	-12.0115		2.0485
36-40 years old	23-30 years old	9.18947^*^	2.23557	.000	3.3834		14.9955
	31-35 years old	9.74464^*^	2.66376	.002	2.8265		16.6627
	41-50 years old	4.76316	2.92592	.366	-2.8358		12.3621
41-50 years old	23-30 years old	4.42632	2.28674	.218	-1.5126		10.3652
	31-35 years old	4.98148	2.70684	.259	-2.0485		12.0115
	36-40 years old	-4.76316	2.92592	.366	-12.3621		2.8358
By Years of Practice							
1-2 years	3-4 years	.76316	1.93316	.979		-4.2575	5.7838
	5-10 years	-2.11765	1.99829	.714		-7.3075	3.0722
	>10 years	-6.11765^*^	1.99829	.014		-11.3075	-.9278
3-4 years	1-2 years	-.76316	1.93316	.979		-5.7838	4.2575
	5-10 years	-2.88080	2.14690	.538		-8.4566	2.6950
	>10 years	-6.88080^*^	2.14690	.009		-12.4566	-1.3050
5-10 years	1-2 years	2.11765	1.99829	.714		-3.0722	7.3075
	3-4 years	2.88080	2.14690	.538		-2.6950	8.4566
	>10 years	-4.00000	2.20573	.271		-9.7285	1.7285
>10 years	1-2 years	6.11765^*^	1.99829	.014		.9278	11.3075
	3-4 years	6.88080^*^	2.14690	.009		1.3050	12.4566
	5-10 years	4.00000	2.20573	.271		-1.7285	9.7285

## Discussion

The present study investigates EM physicians' opioid prescribing behavior and determines which factors lead to their different prescribing patterns. It is interesting to know how EM physicians handle their decisions in OA prescriptions against the increasingly high prevalence of OA morbidity and mortality rates. As per our knowledge, this is the first study in Saudi Arabia that investigates the behavior of EM physicians toward opioid prescription which is an important field given the high prevalence of drug addiction and dependence.

The data in this study suggest that increasing physicians’ age and increasing years of practice were associated with more distinct prescribing behavior, with no significant difference in regard to the level of practice or gender. The variation was most obvious between physicians at the age of (30-35 years) and their senior group (36-40 years). Expectedly, we found out that the level of prescribing behavior differs also between junior and senior residents. In a study done by Leventhal et al., non-EM training program residents prescribed higher amounts of opioid medication than EM residents who attended the curriculum [9]. In another study done in the USA, performance on knowledge exams is associated with clinically meaningful prescribing behavior, as physicians who score well on these examinations may be more responsive to changes in the standards of care [10]. As it was emphasized by Sinnenberg et al, the decision to prescribe opioids to patients in the ED settings is complex to the degree that pushed physicians to seek guidance outside of their departmental policy [11]. Accordingly, the solution-based strategies should target these determinants directly using evidence-based approaches that reach beyond the preset empirical guidelines and general educational points. Shared decision-making strategies and patient-facing decision aids are likely to diminish the tension experienced in challenging situations [12].

Regarding the specific determinants of physician OA prescribing behavior, our data indicate that physicians are naturally more willing to prescribe OA solely based on apparent pain distress. They also tend to prescribe them when they realize that the number of medications given is not enough to control the pain. Other major determinants were understanding of the cause of the pain in addition to the objective parameters which are the patient’s reported pain score, vital signs, and physical exam results. However, physicians' hesitation to prescribe OA increased whenever there were concerns about its side effects and possible substance abuse/dependence among their patients. 

On the other hand, we have learned that our physicians were unlikely to be influenced by their colleague’s prescribing culture or by their previous experiences with this type of medication. Also, the physicians in this study do not appear to be preoccupied with the ideation of patients’ malingering. Surprisingly, the last two factors mentioned were equally marked throughout all of our physicians’ groups which likely indicates unified and objective pain control parameters. The situation was mildly different in the study of Pomerleau et al. [13]. Their investigations found that the attendings were not affected by their cultures whereas residents and advance practitioners were affected, although it was only to a limited degree. In their results, the highest decisional factors were patients’ opioid prescription history, substance abuse history, provisional diagnosis, clinical gestalt, and provider’s concern about unsafe use of the medication. On the other hand, the lowest regarded factors were patients’ age, satisfaction, and the prescribing culture of the clinical site as we mentioned earlier.

Continuous prescription of opioid medications is tantamount to drug addiction. Our physicians showed the highest concerns about the over-utilization of these medications. In this scenario, physicians tend to look for the patient’s opioid prescription history and reputation of drug addiction before deciding to prescribe OA. In a study published by The University of Texas in the USA, two-thirds of the physicians reported that they were “somewhat willing” to “extremely willing” to prescribe long-acting opioids to their patients with chronic nonmalignant pain (CNMP) [14]. However, unwilling physicians held stronger beliefs that prescribing opioids would lead to patient abuse, addiction, and regulatory scrutiny compared to willing physicians. In this regard, Hartmann et al. emphasized the implementation of a guideline for the prescribing of restricted medications as they practically succeeded in decreasing the issued OA down to 31% [15]. We believe that as the prevalence of addiction is unnecessarily correlated with the reduced amount of OA prescriptions, this remarkable reduction definitely scaffolds future guidelines for better utilization of these medications.

Our study results have been possibly affected by multiple factors. Recall bias is likely since some clinicians’ answers might not reflect their exact daily clinical practice. Also, as in many survey-based studies, there was some response and non-response bias. Regardless that we included multiple centers in our population, a more comprehensive study with a larger population size should be conducted for the most validated results.

## Conclusions

Overall, EM physicians demonstrated comparable prescribing behavior among their different categories. The behavior varies significantly with the progression of physicians’ age and years of experience while the other factors did not show significant variability. Specifically, influencing factors were seeing patients who were distressed due to pain, the number of medications that were given to control the pain, and patients’ reported pain scores while unwilling to prescribe could be due to a history of drug abuse and the possible medication side effects. Further practical guidelines, departmental policies, and clinical research are required to improve this critical aspect of every emergency department.
